# Is povidone-iodine pleurodesis as effective, safe and well tolerated as talc pleurodesis for recurrent malignant pleural effusions?

**DOI:** 10.1093/icvts/ivad192

**Published:** 2024-01-16

**Authors:** Sophie A Bonser, Michael Z L Zhu, Glenn S McKay

**Affiliations:** Department of General Surgery, Royal Perth Hospital, Perth, WA, Australia; Department of Cardiothoracic Surgery, The Canberra Hospital, Canberra, ACT, Australia; Department of Cardiothoracic Surgery, The Canberra Hospital, Canberra, ACT, Australia

**Keywords:** Malignant pleural effusion, Pleurodesis, Povidone-iodine, Talc

## Abstract

A best evidence topic in thoracic surgery was written according to a structured protocol. The question addressed was ‘For patients with malignant pleural effusion is chemical pleurodesis with povidone-iodine as effective, safe and well tolerated as talc pleurodesis for prevention of recurrent malignant pleural effusions?’. A total of 124 papers were found during the search, of which 8 represented the best evidence to answer the clinical question. The authors, journal, date, country of publication, patient group studied, study type, relevant outcomes and results of these papers were tabulated. At present, medical-grade talc is the most commonly used agent for chemical pleurodesis due to its high success rate, extensive history of clinical use and well-known side-effect profile. However, studies using povidone-iodine seek to establish it as a readily available,low-cost alternative to talc that can be easily administered through an intercostal catheter at the bedside. The summation of available evidence suggests that povidone-iodine is a safe, well-tolerated and equally efficacious agent for pleurodesis in the setting of malignant pleural effusion, when compared to talc.

## INTRODUCTION

A best evidence topic was constructed according to a structured protocol. This is fully described in the *ICVTS* [1].

## THREE-PART QUESTION

For [patients with malignant pleural effusion], is [chemical pleurodesis with povidone-iodine] as safe, effective and well tolerated by patients as [talc pleurodesis] for [prevention of recurrent pleural effusions]?

## CLINICAL SCENARIO

A 75-year-old male, who 5 years prior had radical right upper lobectomy for primary lung adenocarcinoma, presents with increasing shortness of breath. A chest X-ray reveals a large right-sided pleural effusion. You insert an intercostal catheter (ICC) drain and send the pleural fluid for cytology. This returns adenocarcinoma cells, confirming a malignant pleural effusion (MPE) and stage 4 metastatic disease. With the fluid drained the right lung appears fully expanded on repeat chest X-ray. It is thought the patient should have a pleurodesis to prevent recurrent pleural effusions. You are unsure whether chemical pleurodesis with povidone-iodine would be as effective, safe and well tolerated by the patient compared with graded talc pleurodesis. You search the literature for the best available evidence.

## SEARCH STRATEGY

Both Embase and Ovid databases were searched on the 28th of December 2022 using the following MESH search terms:

[pleurodesis.mp] OR [effusion.mp] OR [malig*.mp] AND [talc*.mp] OR [iodine.mp] OR [povidone-iodine.mp] OR [povidone*.mp] OR [iodopovidone.mp] OR [iodo-povidone.mp]. The reference and citation list of relevant articles were searched to identify potential articles not returned by the PubMed search.

## SEARCH OUTCOME

A total of 121 papers were found using the reported search. Conference abstracts, single-agent, non-comparative studies and those not referencing MPE were excluded. Two papers were identified from the referenced works and a further was published during the time of writing totalling 124 papers. From these, 8 papers were identified that provided the best evidence to answer the question (Table 1).

**Table 1:** Best evidence papers

Author, date, journal, country, Study type (level of evidence)	Patient group	Outcomes	Key results	Comments
Dipper *et al.*, (2020), Cochrane Database of Systematic Rev, UK [2] Meta-analysis (level I)	Databases: CENTRAL, Medline (Ovid), Embase (Ovid). Inclusions: RCTs of intrapleural interventions for adults with symptomatic MPE. A: Povidone-iodine via ICC post-VATS (*I*) versus VATS talc insufflation (*TP*) Articles (*n* = 1) Participants (*n* = 42; *I *=* *20, *TP *=* *22) B: Povidone-iodine via ICC versus talc slurry via ICC (*TS*) Articles (*n* = 2) Participants (*n* = 75; *I *=* *36, *TS *=* *39)	Pleurodesis failure	A: OR 1.76 [0.26, 11.8], *P *=* *0.56 B: *I* 15.0% (3/20) vs *TP* 9.1% (2/22) A: OR 1.17 [0.32, 4.25], *P *=* *0.81 B: *I* 16.7% (6/36) vs *TS* 15.4% (6/39)	This network meta-analysis also examined talc against other available methods of pleurodesis including bleomycin and tetracyclines. Studies included: - Mohsen - Agarwal - Ibrahim Conclusions: Local availability, experience, adverse events and patient preference should be considered when selecting intervention.
		Fever	A: OR 0.24 [0.02, 2.33], *P *=* *0.22 B: *I* 5.0% (1/20) vs *TP* 18.2% (4/22) A: OR 0.93 [0.28, 3.13], *P *=* *0.91 B: *I* 16.7% (6/36) vs *TS* 17.9% (7/39)	
		Pain A: Pain requiring NSAIDs or narcotics B: Any pain	A: OR 0.10 [0.01, 1.99], *P *=* *0.13 B: *I* 0% (0/20) vs *TP* 18.2% (4/22) A: OR 0.5 [0.14,1.83], *P *=* *0.29 B: *I* 75.0% (27/36) vs *TS* 82.1% (32/39)	
Muthu *et al.*, (2021), Supportive Care Cancer, Online [3] Meta-analysis (level I)	Databases: PubMed, Embase Inclusions: >10 participants per study receiving povidone-iodine pleurodesis for MPE with efficacy outcomes available. Exclusions: conference abstracts, editorials, reviews, case reports, <10 patients. Observational (*n* = 15) Comparison of povidone-iodine (I) versus talc (T) (*n* = 2) RCT (*n* = 11) Comparison of povidone-iodine (I) versus talc (T) (*n* = 4)	Success	I versus T: pooled RR of 0.97 [CI 0.85-1.11], *P* = 0.68	This network meta-analysis also examined povidone-iodine against bleomycin, cyclophosphamide, doxycycline, 5-fluorouracil, tetracycline, vincristine and viscum. Studies included: - Mohsen - Agarwal - Shouman - Ibrahim - Das Conclusion: No statistical difference between povidone-iodine and the comparative agents in terms or success or safety.
Agarwal *et al.*, (2011), Respirology, India [4] RCT (level II)	I: Povidone-iodine via ICC (*n* = 18) T: Talc slurry via ICC (*n* = 18) Inclusions and exclusions as per Dipper *et al.*, meta-analysis	Failure	I*:* 5.5% (1/18) T: 11.1% (2/18)	Conclusion: Povidone-iodine and talc are equally efficacious and safe.
		CP	I: 100.0% T: 100.0%	
		ARDS	I: 0.0% T: 0.0%	
		Fever	I: 10.3% T: 14.7%	
		Systemic hypotension	I: 0% T: 0%	
Mohsen *et al.*, (2011), Eur J Cardiothorac Surg, Egypt [5] RCT (level II)	Inclusion: MPE due to metastatic breast cancer. I: Povidone-iodine via ICC post-VATS (*n* = 20) T: VATS talc insufflation (*n* = 22) Mean follow-up: 22.6 months (range: 8–48)	CR PR Failure	I: 85.0% (17/20) T: 86.4% (19/22) I: 0.0% (0/20) T: 4.5% (1/22) I: 15.0% (3/20) T: 9.1% (2/22) *P *=* *0.9	Conclusion: Povidone-iodine is a good alternative to talc in MPE due to metastatic breast cancer. Povidone-iodine is available, cost effective, safe, can be given through a ICC and can be repeated if necessary
		MRC dyspnoea scale I MRC dyspnoea scale II	I: 75.0% (15/20) T: 63.6% (14/22) *P *=* *0.92 I: 25.0% (5/20) T: 36.4% (8/22) *P *=* *0.79	
		Pain requiring NSAIDs or narcotics	I: 0.0% (0/20) T: 18.2% (4/22) *P *=* *0.2	
		Fever	I: 5.0% (1/20) T: 18.2% (4/22) *P *=* *0.5	
		Mean postoperative stay (days)	I: 4.5 ± 1.1 T: 5.7 ± 2.0 *P *=* *0.02	
		Mean survival (months)	I: 33.8 T: 27.7 *P *=* *0.2	
Shouman *et al.* (2012), Egypt J Chest Dis Tuberc, Egypt [6] RCT (level II)	Inclusions: MPE Massive pleural effusion or rapidly accumulation. Subjective improvement in dyspnoea following thoracocentesis. Total re-expansion of lung after fluid drainage. Pleural fluid pH >7.2. Exclusions: Atelectasis due to endobronchial obstruction. Pleural fluid pH <7.2. Prior intrapleural therapy. Any hemithorax radiotherapy. I: Povidone-iodine* (*n* = 15) T: Talc slurry 5 g (*n* = 15) * 10%, 20 ml diluted with 80 ml normal saline Via ICC	CR	At 30 days I: 66.7% (10/15) T: 80.0% (12/15) At 60 days I: 60.0% (9/15) T: 73.3% (11/15)	This study compared tetracycline, talc slurry, povidone-iodine and bleomycin. Conclusions: No agent was significantly better than others. All were better than control group.
		CP worse post-procedure	I: 13.3% (2/15) T: 26.7% (4/15)	
		Fever	I: 33.3% (5/15) T: 26.7% (4/15)	
		Dyspnoea worse than pre-procedure	I: 6.7% (1/15) T: 33.3% (5/15)	
Ibrahim *et al.*, (2015), J Cardiothorac Surg, Egypt [7] RCT (level II)	Inclusions: Clinical and histopathological confirmation of recurrent MPE. Exclusions: Povidone-iodine allergy. Incompletely inflated lung on radiograph. I: Povidone-iodine (*n* = 18) T: Talc pleurodesis (*n* = 21) Both through ICC	Pleurodesis response	CR I: 66.7% (12/18) T: 71.4% (15/21) PR I: 5.6% (1/18) T: 9.5% (2/21) Failure I: 27.8% (5/18) T: 19.0% (4/21) *P *=* *0.20	Conclusions: Povidone-iodine is a good alternative to talc. It is available, cost effective, safe and repeatable.
		Pain	None I: 50.0% (9/18) T: 33.3 % (7/18) Mild I: 50.0% (9/18) T: 57.1% (12/21) Moderate/severe I: 0.0% (0/18) T: 9.5% (2/21) *P *=* *0.29	
		Fever	I: 19.2% (4/18) T: 22.3% (4/21) *P *=* *0.807	
		Length of stay	I: 4.7 ± 1.2 T: 4.2 ± 1.0 *P *=* *0.17	
Das *et al.*, (2008), J Indian Med Assoc, India [8] Observational study (level III)	Inclusions: MPE confirmed + dyspnoea. Life expectancy >4 weeks. Clinical + radiological evidence of mediastinal shift to the opposite side of pleural effusion. Relief of dyspnoea with therapeutic thoracentesis. Exclusions: Asymptomatic pleural effusion. Mediastinum central or shifted to the ipsilateral side. Life expectancy <4 weeks. Chylothorax. Pleural effusion secondary to chemosensitive malignancy. Known hypersensitivity to sclerosing agents. I: Povidone-iodine (*n* = 28) T: Talc slurry (*n* = 24)	CR	I: 86.0% (*n* = 24) T: 79.0% (*n* = 19)	Conclusion: Both are equally effective and safe. Povidone-iodine could be preferred due to availability and low cost.
		PR	I: 4.0% (*n* = 1) T: 12.0% (*n* = 3)	
		Failure	I: 11.0% (*n* = 3) T: 8.0% (*n* = 2)	
		CP	I: 17.0% (*n* = 5) T: 16.0% (*n* = 4)	
		Fever	I: 11.0% (*n* = 3) T: 12.0% (*n* = 3)	
Nistor *et al.*, (2014) FARMACIA, Romania [9] Observational study (level III)	Inclusions: Patients admitted with diagnostic established MPE. Exclusions: Known thyroid disease I: Povidone-iodine 2% (*n* = 46) T: Talc powder 5 g (*n* = 39) Gender: M (*n* = 40) F (*n* = 45)	Pleurodesis response	Complete response I: 76.1% (35/46) T: 76.9% (30/39) Partial response I: 19.6% (9/46) T: 17.9% (7/39) Failure I: 4.3% (2/46) T: 5.1% (2/46)	Conclusion: Chemical pleurodesis with povidone-iodine is a safe procedure with high therapeutic efficiency and lower complications than talc powder.
		Fever	I: 4.3% (*n* = 2) T: 64.1–% (*n* = 25)	
		Thoracic pain	I: 26.1% (*n* = 12) T: 10.2% (*n* = 4)	
		Dyspnoea	I: 4.3% (*n* = 2) T: 5.1% (*n* = 2)	
		PE	I: 0.0% (*n* = 0) T: 2.6% (*n* = 1)	
		Empyema	I: 0.0% (*n* = 0) T: 2.6% (*n* = 1)	

Abbreviations: ARDS: Acute Respiratory Distress Syndrome; CP: Chest Pain; CR: Complete Response (nil recurrence of effusion); ICC: intercostal chest catheter; MPE: malignant pleural effusion; MRC: Medical Research Council; NSAIDs: non-steroidal anti-inflammatory; OR: odds ratio; PE: pulmonary embolism; PR: Partial Response (Recurrence of fluid, successfully managed with re-administration); RCTs: randomized controlled trials; VATS: video-assisted thoracoscopic surgery.

## RESULTS

Of the 8 articles included, 2 articles were meta-analyses, 5 were randomized controlled trials (RCTs) and 2 were observational studies. Articles were published between 2008 and 2021.

The 2020 Cochrane review by Dipper *et al.* [2] is most often cited when recommending talc, providing an extensive review of all available sclerosing agents. The review culminates in 80 articles and 5507 total participants, the largest participant population to date. Despite such an evidence body, only 3 articles specifically compared povidone-iodine and talc sclerosis reducing the pertinent comparison to only 117 participants. Promisingly, povidone-iodine was identified as a potentially efficacious agent and was ranked 7/21 only falling behind various versions of talc, manual pleurodesis and mepacrine pleurodesis. However, the authors identified several limitations in these studies, namely significant heterogeneity, observational nature and inconsistencies in adverse event reporting. Thus, rather than a definitive recommendation, the authors conditionally recommended graded talc as the sclerosing agent of choice administered via thoracoscopic poudrage.

Muthu *et al.* [3] published a systematic review and meta-analysis in 2021. It again challenges the enthusiasm for talc citing expense, limited availability, and risks of respiratory failure. The review included a total of 26 papers(11 RCTs and 15 observational studies) and focused on the safety and efficacy profile of povidone-iodine to manage MPE. In this meta-analysis, only 4 RCTs and 2 observational studies directly compared povidone-iodine to talc (either poudrage or slurry). Despite a favourable pooled success rate of 90% from 648 povidone-iodine procedures, the review failed to establish any agent, including talc, as statistically superior in terms of safety or efficacy to achieve successful pleurodesis. Concerns that povidone-iodine may cause retinal toxicity or thyroid dysfunction were not observed.

The first level II evidence paper is the largest RCT at the time of writing. Published by Agarwal *et al.* [4], it exclusively focused on povidone-iodine and cosmetic talc predominantly via ICC administration. The Indian-based authors faced restrictionsin obtaining medical-grade talc both due to its significant cost and limited availability. Furthermore, the authors stated that access to thoracoscopic administration, as recommended by Cochrane [2], can be limited in certain health settings. Thus, an alternative was required. The study included 73 patients (iodine: 39 patients versus talc: 34 patients) and compared the efficacy of each agent for both MPE and pneumothoraces. Patients with MPE experienced a high success rate with both agents (povidone-iodine 84.2%, talc 78.9%, respectively). Anecdotally, povidone-iodine was reported to be easier to administer via ICC than talc and has potential cytotoxic benefits compared with talc. Povidone-iodine is universally manufactured to standard specifications while talc in this locality can be both cosmetic and sterile and has variations in particle size. A particular benefit to this RCT is the in-depth evaluation of adverse effects not available in the former papers. Chest pain was universal, fever rates were low but comparable. Significant adverse effects were rare. This thorough adverse-effect analysis begins to ameliorate the caution advised by Cochrane [2] regarding the limited safety profiles of non-talc agents. Ultimately, the authors concluded the agents were equally efficacious and safe.

Mohsen *et al.* [5], conducted a smaller RCT in Egypt comparing povidone-iodine and talc exclusively in MPE patients (20 vs 22, *n* = 44). At the time of writing, talc was not approved for medical use in Egypt. In this RCT, all patients firstly underwent a diagnostic video-assisted thorascopy (VATS) and drainage of pleural effusion. They then either received talc poudrage administered at the time of the diagnostic VATS, or povidone-iodine at the bedside via an ICC.Both subjective and objective minor adverse effects were reported however significant adverse outcomes were not assessed. The study established ICC povidone-iodine as equally efficacious compared with the standard of treatment of talc poudrage via thoracoscopy. Furthermore, there was a statistically significant reduction in hospital stay of 4.5 days in the povidone-iodine group vs 5.7 days in the talc group. The authors concluded the combination of ease of repeatability and administration and its cost-effectiveness made povidone-iodine a suitable and safe agent. However, these are purely procedural elements and the study fails to assess whether ICC administration of talc would result in similar outcomes.

Remedying this administration discrepancy, Shouman *et al.*’s [6] 2012 RCT reached a similar conclusion with the benefit of using ICC administration exclusively. In this study, 4 agents are compared: tetracycline, talc slurry, povidone-iodine and bleomycin against a control with ICC insertion only. Of the 75 patients included, 15 received talc and 15 received povidone-iodine. Unlike the Cochrane review, the effectiveness was not found to be statistically different between any agent. Dyspnoea appeared to be more common in the talc group, while fevers occurred more in the povidone-iodine group (*P *>* *0.05). Chest pain was universal but apparently less among the povidone-iodine group (*P *>* *0.05). Unsurprisingly, all were significantly more effective than placebo alone. Given the consistent efficacy and side-effect profile, the authors recommended that cost and accessibility should be the primary determinants when choosing an agent.

Similar recommendations were reported in the final available RCT by Ibrahim *et al.* [7]. Talc and povidone-iodine alone were compared (talc: 21 patients vs iodine: 18 patients). Efficacy was statistically similar as were the common adverse side-effect profiles. Pain ranged from mild to moderate, but no severe cases were reported. Dyspnoea and fever rates remained under 30% in both groups. Unlike the Mohsen *et al.*, trial, the length of stay did not vary between groups.

Das *et al.* [8], conducted the earliest included prospective observational study of 52 patients with MPE. After initial pleural drainage via an ICC, talc slurry was administered for 24 patients and 28 received povidone-iodine. The scope of questions answered is narrow compared with the previously analysed papers. The only complications assessed were fever and chest pain, neither of which was statistically. Efficacy rates were similar. Despite not reviewing major side effects, the authors concluded that both talc slurry and povidone-iodine are safe and effective agents for pleurodesis in MPE but preferred povidone-iodine due to its global availability and lower cost.

Nistor *et al.* [9], published a single-centre observational study in 2014. A total of 46 patients received povidone-iodine and 39 received talc via thoracoscopy or ICC. The rates of successful pleurodesis and major adverse effects were recorded. Complete response was observed in >76% of cases in both groups with similar partial response of 19.6% (povidone-iodine) and 17.9% (talc). Significant adverse effects were studied including empyema and pulmonary embolism both of which occurred once in the talc group. Rates of pain and dyspnoea were universally low. The authors found talc was highly effective regardless of administration method though agreed with the Cochrane [2] conclusion that talc poudrage is ultimately superior. The small size of this study makes it underpowered to give definitive recommendations but allowed the authors to conclude that povidone-iodine pleurodesis is a safe and efficacious procedure with the appearance of lower complication rates. Further, povidone-iodine is an antiseptic and has a theoretical benefit of reducing infection.

Debate continues regarding the best agent for pleurodesis due to significant limitations with current evidence. Sample size, level of evidence, heterogeneity of sclerosing agents used or methods of administration were noted thus the generalisability of the recommendations is hindered. Despite these limitations, talc poudrage has been widely accepted as the standard of treatment. However, some concerns remain. In early studies, talc had been associated with severe adverse events limiting its approval in several countries. Furthermore, medical-grade talc is expensive and thoracoscopy as a procedure is usually limited to specialist centres with a thoracic surgery service. Thus, the current ‘standard of care’ is reserved for a select population which warrants the need for further research.

The studies included in this BET were similar in their patient inclusion criteria and were limited to patients with a diagnosis of MPE. Themost commonly studied complications included fever, dyspnoea and chest pain. Anecdotal concerns regarding the potential effect of povidone-iodine on pre-existing thyroid disorders were not established. Major complications such as ARDS, empyema and systemic hypotension were inconsistently included.

The Cochrane review [2] was the only article to recommend talc over other agents, primarily due to the availability of extensive background literature. Indeed, the authors stated that the clinical and patient context should be considered prior to choosing an agent. All remaining articles agreed that povidone-iodine was as equally safe, efficacious and more widely available and lower cost compared to talc.

## CLINICAL BOTTOM LINE

This review highlights that the current standard of care for pleurodesis, in the setting of MPE, is based on a limited foundation of evidence and is not universally transferable to all clinical settings such as regional and remote centres or developing countries where access to medical-grade talc may be limited. The summation of available best evidence suggests that povidone-iodine is a safe, well-tolerated andequally efficacious agent for achieving palliative pleurodesis in the setting of MPE. Furthermore, its low cost, accessibility and ease of administration provide important advantages over talc in certain clinical settings.

## Data Availability

All relevant data are within the manuscript and its Supporting Information files.
